# Two-dimensional nanofluid flow impinging on a porous stretching sheet with nonlinear thermal radiation and slip effect at the boundary enclosing energy perspective

**DOI:** 10.1038/s41598-023-32650-0

**Published:** 2023-04-04

**Authors:** Zeeshan, Ilyas Khan, Syed M. Eldin, Saeed Islam, M. Uzair Khan

**Affiliations:** 1 Department of Mathematics and Statistics, Bacha Khan University, Charsadda, KP Pakistan; 2 Department of Mathematics, College of Science Al-Zulfi, Majmaah University, Al-Majmaah, 11952 Saudi Arabia; 3 Center of Research, Faculty of Engineering, Future University in Egypt, New Cairo, 11835 Egypt; 4 Department of Mathematics, Abdul Wali Khan University Mardan, Mardan, KP 25000 Pakistan

**Keywords:** Energy science and technology, Engineering, Materials science, Mathematics and computing

## Abstract

In the current analysis, we examine the heat transmission analysis of nanofluid (NF) movement impinging on a porous extending sheet. The influence of nonlinear thermal radiation (TR), buoyancy force, and slip at the boundary are also examined. The leading partial differential equations (PDEs) are altered to convectional differential equation (ODEs) by suitable transformation. The ODEs are then transformed to first order by introducing the innovative variables and elucidated numerically using bvph2. The Skin Friction (SF) and Nusselt number (NN) are elaborated in detail for Al_2_O_3_, Cu, and TiO_2_ nanoparticles. For validation of the code, ND-solve approach is also applied. The novelty of the current effort is inspect NF flow with heat transfer over extending sheet enclosing thermal and slip effect at the boundary numerically. The thickness of boundary layer increases as the temperature and radiation factors are increased. It is perceived that the fluid velocity decays with the growing values of volume fraction parameter. When permeability and velocity slip parameters are improved the velocity outline enhances. It is investigated that the temperature inside the fluid enhances as the values of velocity slip factor, permeability factor and Biot number are augmented. For the growing values of temperature ratio, volume friction, and thermophoresis factor the temperature is enhances. It is detected that the slip factor causes the friction factor to decrease. Furthermore, the existent study is associated with the preceding.

## Introduction

NF technology development is a very important study in mathematics, manufacturing, physics, and materials science. Architects and researchers strive to effectively convey adequate understanding about heat transfer process in NF for most applications of practical interest. In automotive industry, NFs can be used in engines to improve their efficiency and reduce emission. By enhancing the thermal conductivity of engine oil, NFs can improve heat transfer, reducing engine wear and tear and improving fuel efficiency. This can lead to reduced emission and a longer lifespan for the engine. NFs have a wide of potential applications in various industries including electronics, energy production, and automotive engineering. Their ability to improve thermal conductivity and heat transfer efficiency can lead to improved performance, reduced energy consumptions, and increased lifespan for machinery and devised. Choi^1^ developed a creative and novel technology process that needs the adding of materials nano-size particles and the ordinary fluid for rapid heat flow with higher rate of thermal conductivities effect in terms of heat energy and environmental cleanup. Due to their prominently improved thermos-physical nature, NFs serve as ideal coolants in much considerable manufacturing utilization. Buongiorno^2^ considered notable features of thermophoretic diffusion and the Brownian movement effect around the molecules of the NF. Afterward, mathematician and investigators have extensively used NFs to scrutinize the real-world problems like manufacturing uses^3,4^, biomedical production^5^, and solar thermal solicitations^6^ and to several other making fields functional for diverse physical problems^7–25^. Motsumi et al.^7^ considered the inspiration of viscid dissipation and thermal characteristics of NF passed through a porous flat plate. Saidur et al.^8^ gave the comprehensive review on NF flow. The application of NF and its feature is investigated by Wong et al.^9^. Sandeep and Ashwinkumar^10^ explored the MHD flow of stagnation point flow containing nanoparticles using Carreau fluid. Similarly, Sandeep et al.^11^ discussed the impact of hybrid NF with TR. Samrat et al.^12^ investigated the heat transfer (HT) in hybrid NF and gave the simultaneous solutions. Some application of NF applied for different physical problems were studied in^13–25^.

Convection boundary layer fluid above a stretched surface affected by thermal radiation has a wide range of industrial applications, such as carbonization, heating system design, nuclear reactor protection, fluidization heat pumps, solar reservoirs, solar thermal collectors, photochemical reactors, and many more. Many activities in designing take place at high temperatures, making understanding of heat transfer by radiation critical for the designing of the relevant equipment. Such engineering domains include nuclear power plants, steam turbines, and different engine technologies for airplanes, rockets, spacecraft, and communications satellites. Many scientists investigated the influence of radiative on heat transport of Newtonian and non-Newtonian fluids over stretched surfaces in light of in MHD 3D Jeffrey NF under the inspiration of non-linear TR. this applicability. Pantokratoras^26^ used a novel radiation factor called film radioactive factor to examine the influence of Rosseland model both in term of linear and linear form on natural convection down a vertically homogeneous plate for the very first time in his study. Cortell^27^ addressed liquid motion and stochastic TR transfer through a stretched sheet. Mushtaq et al.^28^ investigated stochastic radioactive heat exchange in a Williamson fluid caused by solar light. Laxmi et al.^29^ and shehzad et al.^30^ examined the impact of nonlinear TR and a consistent convinced magnetic field in 3D movement of NF in the involvement of thermophoresis and Brownian movement inspirations.

For the majority of non-Newtonian models, the no-slip requirement is insufficient because certain polymer liquefies frequently reveal tiny wall slip that is controlled by a non-linear and monotonous correlation amid the slip flow and the adhesion. Once the liquefied is particulate, such as delays, spumes, and polymerization resolutions, small velocity slip may occur on the boundary of stretching sheet. The slip properties can occur at the boundaries of tubes, walls, curled surfaces, etc., in a variety of industrial processes. The Navier velocity slip condition is a common method for examining slip occurrences. When refining prosthetic heart valves and interior cavities, a slip boundary layer flow issue occurs. For boundary layer movement and heat transport caused by a stretched surface, authors recently achieved analytical and numerical solutions. Aziz^31^ explored the magnetized movement and rate of heat diffusion of a nanomaterial fluid via an absorptive surface with slip at the wall. Pantokratoras et al.^32^ scrutinized the heat and mass transmission in a 2D electrically conducting slippage movement of an unsteady laminar, alumina water-based micro-channel flow across a flat surface. Goyal et al.^33^ has offered a thorough analysis of the issue of mixed-convective movement and heat exchange of Maxwell-fluid passed a porous stretchable sheet. Nadeem et al.^34^ studied the influence of slip happening in MHD natural convective movement of Nano sized particles-fluid across a movable surface via Lie group transformations and computational approaches.

Bejawada et al.^35^ investigated the 2D mixed convection through radiation impact using NF through a persuaded sheet. Shankar et al.^36^ examined the chemical reaction, Soret and Dufour influence on magnetized Casson liquid over a vertical porous stretchable sheet enclosing heat analysis and slip effects. Mishra et al.^37^ provided the arithmetical solution of magnetized williamson NF using the non-darcy model. Impact of slip on transient movement Yanala over a vertical sheet with ramped heat with chemical and TR was explored by Yanala et al.^38^. Reddy et al.^39^ explored the TR and thermal slip effect on magnetized boundary layer movement with heat-mass transportation on Williamson NF over a porous medium. Alipour et al.^40^ observed the hybrid NF over a porous cavity by using response surface method. Akbari et al.^41^ obtained the analytical results for non-Fourier heat transmission inside a hollow sphere. Faghiri et al.^42^ considered the non-Newtonian fluid model with non-uniform wall heat flux through a circular tube. Similarly, Talebi et al.^43^ deliberated the dusty-hybrid nanoliquid movement in permeable channel using RBF approach.

Based on the given literature, the goal of the existing analysis is to investigate the heat transfer analysis of NF flow impinging on a permeable extending sheet. The effect of nonlinear TR, buoyancy force, and slip at the boundary are also examined. The leading partial differential equations are altered to convectional differential equation (ODEs) by suitable transformation. The ODEs are then transformed to first order by introducing the new variables and solved numerically using bvph2. The Skin Friction (SF) and Nusselt number (NN) are elaborated in detail for Al_2_O_3_, Cu, and TiO_2_ nanoparticles. For validation of the code, ND-solve approach is also applied. Furthermore, the current work is equated with the published work.

The novelty of the current effort is to examine the convective heat transportation of nanomaterial fluid in porous matrix on an extending surface in the occurrence of viscous force (buoyancy force) and slip impact at the boundary with nonlinear thermal radiation. So, in some limiting cases the present work is associated with the obtainable literature. The current research has significant implications for advanced manufacturing thermodynamic efficiency, heat exchange enhancement in photovoltaic systems, and biomaterials.

## Mathematical formulation

**Figure 1:**
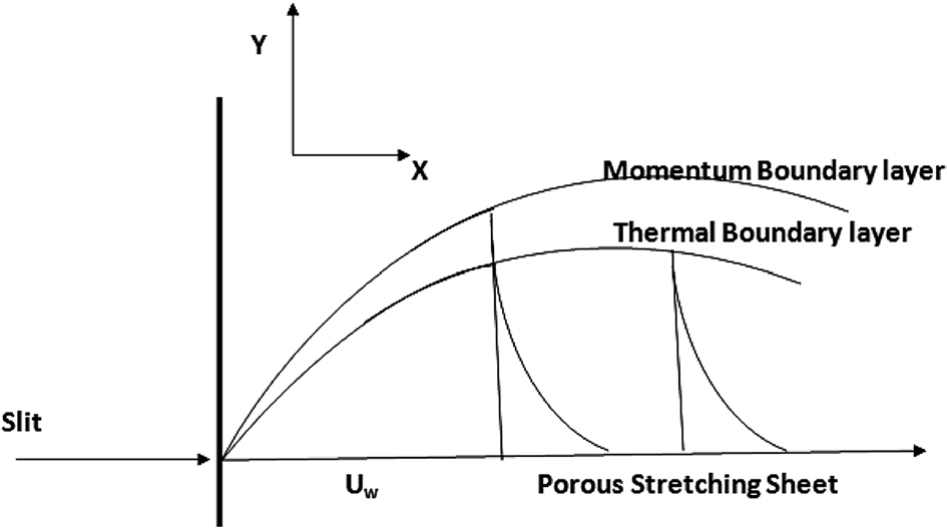
Flow geometry of the problem.

Consider the movement and heat transmission of 2D laminar viscous nanomaterial fluid through a porous stretched surface in a boundary layer flow. The flow is constrained to the plane $y > 0$ and the sheet coincide with the plane $y = 0$. As seen in Fig. 1, the flow is persuaded by the surface being linearly stretched by the coincident application of two-identical and opposing forces along the x-axis. The surface is then stretched while maintaining its initial position with a velocity ${U}_{w}(x) = ax$, while $a > 0$ denotes the rate of stretching and $x$ is measured along the stretched sheet that fluctuates linearly with a distance from the slot. The ambient temperature is denoted by ${T}_{\infty }$. The heat surface transfer is maintained by convective heat transfer ${T}_{f}$. The water-based NF is deliberated for this analysis containing $A{l}_{2}{O}_{3}$ nanoparticle. It is expected that the nanoparticles are homogeneous in size and shape. Additionally, it is presumable that the liquid phase and the nanomaterial are in thermal equilibrium state.

The flow equations for the propose model are^17,27^:

1 $$ \frac{\partial \left( u \right)}{{\partial x}} + \frac{\partial \left( v \right)}{{\partial y}} = 0 $$

2 $$ u\frac{\partial u}{{\partial x}} + v\frac{\partial u}{{\partial y}} = \frac{{\mu_{nf} }}{{\rho_{nf} }}\left( {1 + \frac{1}{\beta }} \right)\frac{{\partial^{2} u}}{{\partial y^{2} }} - \frac{{\upsilon_{nf} }}{{k^{^{\prime}} }}u $$

3 $$ u\frac{\partial T}{{\partial x}} + v\frac{\partial T}{{\partial y}} = \alpha_{nf} \frac{{\partial^{2} T}}{{\partial y^{2} }} + \frac{1}{{\left( {\rho Cp} \right)_{nf} }}\frac{{\partial q_{r} }}{\partial y} $$

Here, $\rho_{nf}$ stands for the density of the fluid containing nanoparticles, $\mu_{nf}$ for NF viscosity, $\alpha_{nf}$ thermal conductivity and $\left( {\rho Cp} \right)_{nf}$ for heat capacity ^14,33^

$$\begin{aligned} \alpha_{nf} = \frac{{k_{nf} }}{{\left( {\rho Cp} \right)_{nf} }}, \\ \rho_{nf} = \left( {1 - \phi } \right)\rho_{f} + \phi \rho_{s}, \\ \rho_{nf} = \left( {1 - \phi } \right)\rho_{f} + \phi \rho_{s}, \\ \mu_{nf} = \frac{{\mu_{f} }}{{\left( {1 - \phi } \right)^{2.5} }}, \\\left( {\rho Cp} \right)_{nf} = \left( {1 - \phi } \right)\left( {\rho Cp} \right)_{f} + \phi \left( {\rho Cp} \right)_{s} \end {aligned} $$

4 $$ \frac{{k_{nf} }}{{k_{f} }} = \frac{{k_{s} + 2k_{f} - 2\phi \left( {k_{f} - k_{s} } \right)}}{{k_{s} + 2k_{f} + 2\phi \left( {k_{f} - k_{s} } \right)}} $$

It should be indicated that the aforementioned expression for ${k}_{nf}$ can only be used with spherical nanoparticles; other shapes of nanoparticles are not taken into account^33^.

The NF ${\mu }_{nf}$ viscosity has also been roughly compared (Brinkman^34^) based on fluid viscosity ${\mu }_{f}$ that contains a diluted suspension of fine spherical particles. Table 1 lists the thermophysical features of the convectional fluid (water) and several nanoparticles.

**Table 1:** Thermal properties of water and nanoparticles.

Thermo-physical prop	$k$	$\rho$	$C_{p}$
Pure water	0.613	997.1	4179
Copper $Cu$	400	8933	385
Titanium Oxide (TiO_2_)	8.9538	4250	686.2
Aluminum Oxide (Al_2_O_3_)	40	3970	765

It is believed that convective heat transfer keeps the sheet surface temperature at a fixed temperature ${T}_{f}$ (see Ref.^34^). The analogous boundary constraints are^30^,

5 $$ \begin{gathered} u = U_{w} (x) + K_{1} \,\frac{\partial u}{{\partial y}}\,,v = 0, - k_{f} \left( {\frac{\partial T}{{\partial y}}} \right) = h_{f} \left( {T_{f} - T} \right),\,\,\,{\text{at }}y = 0, \hfill \\ u = 0,T = T_{\infty } \,{\text{as }}y \to \infty \hfill \\ \end{gathered} $$

We employ the nonlinear Rosseland radiation approximation rather than the linearized Rosseland estimate, after which one may get conclusions for both small and large variations between ${T}_{f}$ and ${T}_{\infty }$.

The radiative heat flux is summarized into the following using the Rosseland approximation (Rosseland,^2,33,34^):

6 $$ q_{r} = \frac{{4\sigma^{*} }}{{3k^{*} }}\frac{{\partial T^{4} }}{\partial y} $$

The heat flux radiation for a boundary layer movement across a straight flat surface is reduced ^14^ as follows:

7 $$ q_{r} = - \left( {\frac{{16\sigma^{*} T_{\infty }^{3} }}{{3k^{*} }}} \right)\frac{\partial T}{{\partial y}} $$

In light of Eqs. (7), (3) becomes

8 $$ u\frac{\partial T}{{\partial x}} + v\frac{\partial T}{{\partial y}} = \alpha_{nf} \frac{{\partial^{2} T}}{{\partial y^{2} }} - \frac{1}{{\left( {\rho Cp} \right)_{nf} }}\left( {\frac{{16\sigma^{*} T_{\infty }^{3} }}{{3k^{*} }}} \right)\frac{{\partial^{2} T}}{{\partial y^{2} }} $$

To simplify the flow governing equations and associated boundary constraints, the succeeding suitable transformations are used:

9 $$ \begin{gathered} u = axf^{\prime}(\eta ),v = - \sqrt {a\nu_{f} } f(\eta ),T = T_{\infty } (1 + (\theta_{r} - 1)\theta (\eta )) \hfill \\ {\text{and }}\eta = \sqrt {\frac{a}{{\nu_{f} }}} y \hfill \\ \end{gathered} $$

where $\theta_{r} = \frac{{T_{f} }}{{T_{\infty } }},\theta_{r} > 1$ is the temperature ratio parameter^21^

It is evident that the similarity variables described in Eq. (9) satisfy Eq. (1). Now, one can obtain by applying Eq. (9) to Eqs. (2) and (8) and to boundary conditions;

10 $$ \left( {1 + \frac{1}{\beta }} \right)f^{\prime\prime\prime} + (1 - \phi )^{2.5} [1 - \phi + \phi \frac{{\rho_{s} }}{{\rho_{f} }}](ff^{\prime\prime} - f^{{\prime}{2}} ) - k_{p} f^{\prime} = 0, $$

11 $$ \frac{{k_{nf} }}{{k_{f} }}\left[ {1 + Nr\left( {1 + \left( {\theta_{w} - 1} \right)\theta } \right)^{3} \theta^{\prime}} \right]^{\prime } + \Pr \left( {1 - \phi + \phi \frac{{\left( {\rho C_{p} } \right)_{s} }}{{\left( {\rho C_{p} } \right)_{f} }}} \right)f\theta^{\prime} = 0, $$

12 $$ \begin{gathered} f\left( 0 \right) = 0\,\,,\,\,f^{\prime}\left( 0 \right) = 1 + Af^{\prime\prime},\,G\left( 0 \right) = 1,\,\,\,\,\theta^{\prime}\left( 0 \right) = Bi\left( {\theta \left( 0 \right) - 1} \right), \hfill \\ \,f^{\prime}\left( \infty \right) \to 1,\,\,\theta \left( \infty \right) = 0. \hfill \\ \end{gathered} $$

Primes indicate differentiation with reverence to $\eta$ in the aforementioned equations.

The SF coefficient ${C}_{f}$ and the local NN $N{u}_{x}$, which are both physical variables of relevance, are defined as

13 $$ C_{f} = \frac{{\tau_{w} }}{{\rho_{f} U_{w}^{2} }}\,,\,\,Nu_{x} = \frac{{xq_{w} }}{{k_{f} \left( {T_{f} - T_{\infty } } \right)}}\left. {\frac{\partial T}{{\partial y}}} \right|_{y = 0} $$

The surface heat flow ${q}_{w}$ and the surface shear stress $\tau_{w}$ are determined by,

14 $$ \tau_{w} = \mu_{nf} \left( {\frac{\partial u}{{\partial y}}} \right), \, q_{w} = - k_{nf} \frac{\partial T}{{\partial y}} + \left( {q_{r} } \right)_{w} {\text{ at }}y = 0 $$

Employ transformation (9), we can write

15 $$ \sqrt {{\text{Re}}_{x} } C_{f} = \frac{1}{{\left( {1 - \phi } \right)^{2.5} }}f^{\prime\prime}(0), \, \frac{{Nu_{x} }}{{\sqrt {{\text{Re}}_{x} } }} = - \frac{{k_{nf} }}{{k_{f} }}\left( {1 + Nr\theta_{w}^{3} } \right)\theta^{\prime}(0) $$

where ${\text{Re}}_{x} = \frac{{ax^{2} }}{{\nu_{f} }}$ is the local Reynolds number.

## Numerical procedure and stability analysis

**Figure 2:**
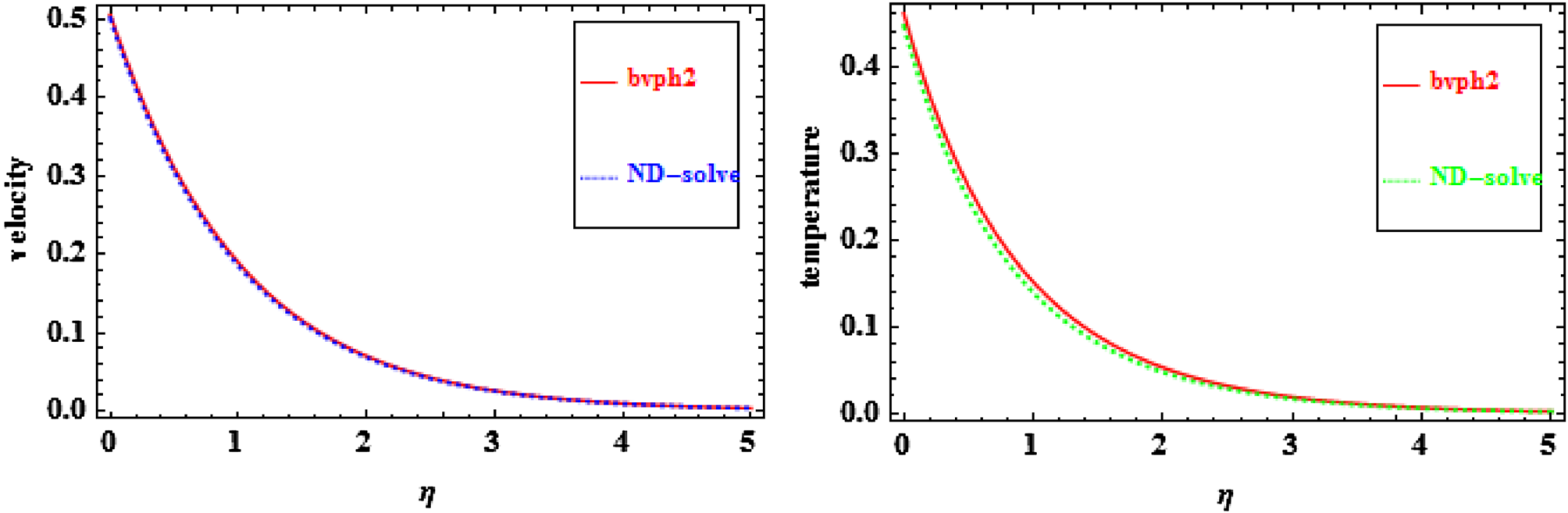
Comparison of bvph2 and ND-solve.

The bvph2 approach has been used to elucidate the scheme of nonlinear equations (Eq. 10) and (Eq. 11) along with boundary conditions (Eq. 12). Equations (10)and (11) corresponding to the boundary conditions are transformed to first order differential equation by introducing the new variables and then resolved numerically. For the confirmation of the results, bvph2 is equated with the ND-solve technique and outstanding settlement is found as shown in Fig. 2. Additionally, the existing work is validated with the former as reported by Goyal et al.^33^ and Laxmi et al.^29^ and excellent settlement is found as shown in Table 2.

**Table 2:** Cssesment of the published work and present work.

$\eta$	Laxmi et al.^29^	Goyal et al.^33^	Current work
1	0.2792	0.2792	0.2793
2	0.6459	0.5649	0.5654
3	0.9225	0.9224	0.9224
4	2.9916	2.9916	2.9962
5	3.4649	3.4649	3.4649
6	3.4649	3.4649	3.4649

## Results and discussion

A theoretically investigated has been done for NTR (nonlinear thermal radiation) of NF (Al_2_O_3_-water) over permeable stretchable surface of velocity slip boundary layer flow under the influence of convective boundary conditions. For the basic fluid, $Pr=10$ is maintained as the Prandtl number. The graphs are sketched for the impacts of the slip factor *A*, volume fraction of nanomaterial factor $\phi$, thermal factor *Nr*, temperature factor ${\theta }_{r}$, Biot amount $Bn$, and porous factor ${P}_{r}$ in order to analyses the properties of velocity filed and temperature profile. The physical explanations for the graphs are also comprehensively explained.

**Figure 3:**
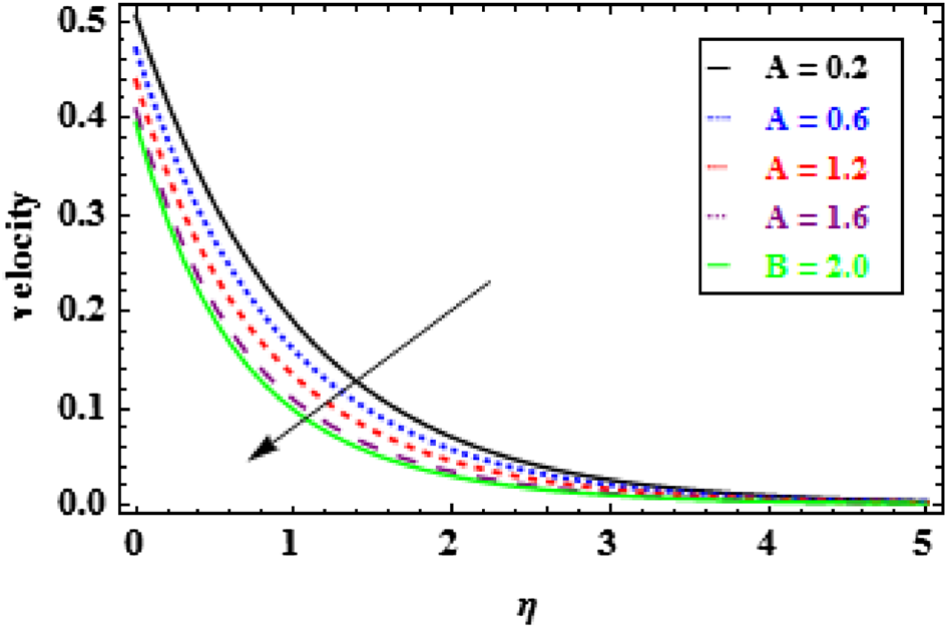
Inspiration of A on velocity.

**Figure 4:**
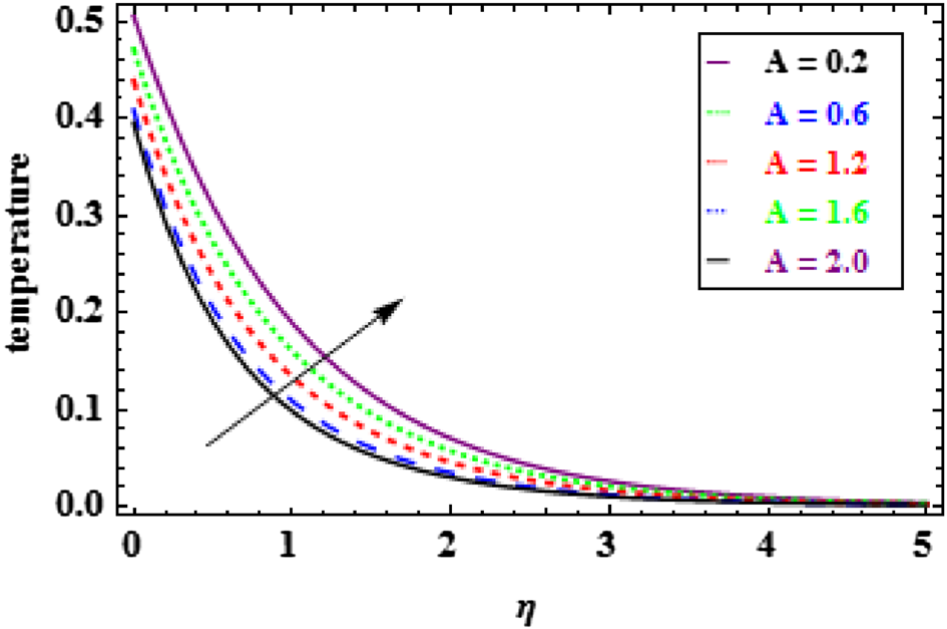
Inspiration of A on temperature.

**Figure 5:**
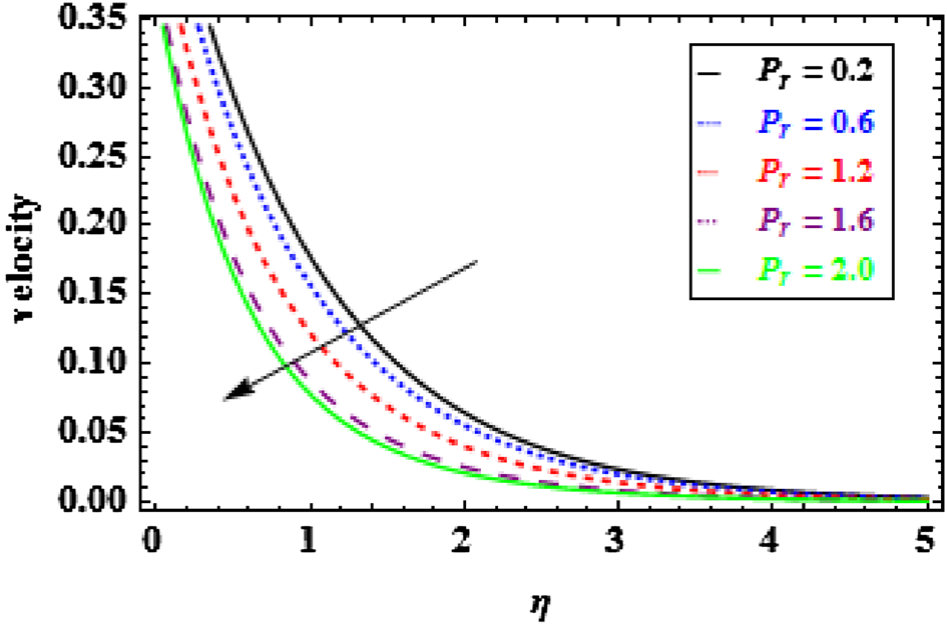
Inspiration of ${P}_{r}$ on velocity.

**Figure 6:**
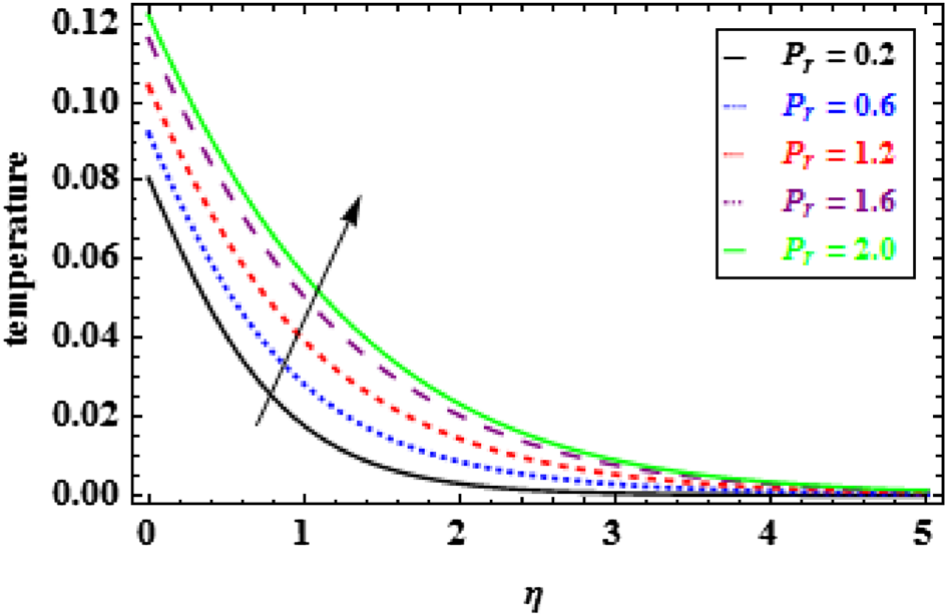
Inspiration of ${P}_{r}$ on temperature.

**Figure 7:**
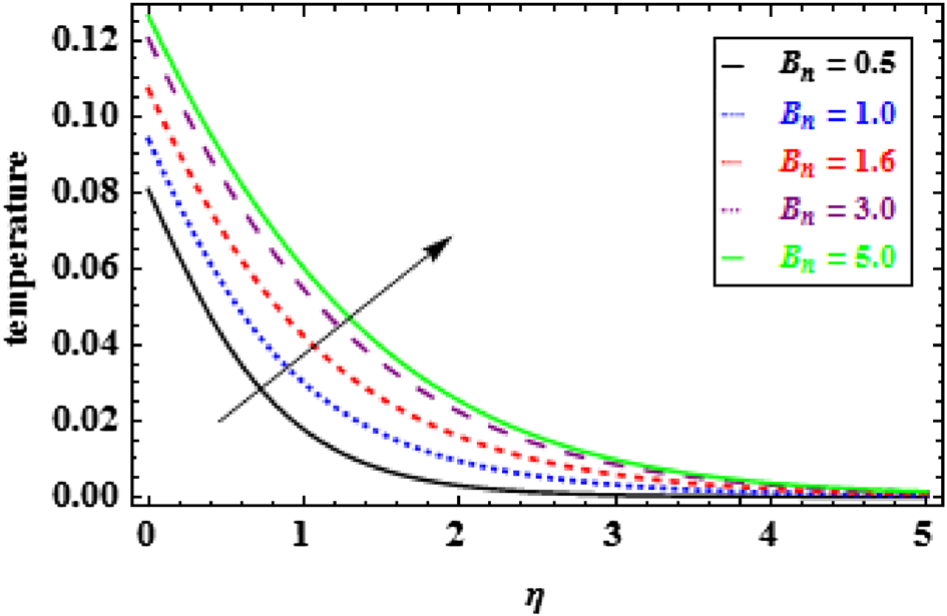
Inspiration of ${B}_{n}$ on temperature.

**Figure 8:**
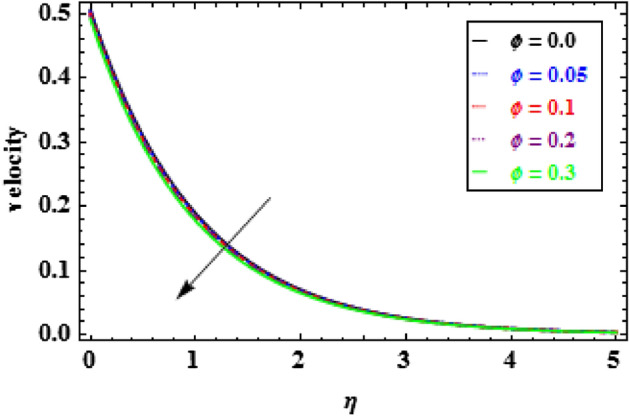
Inspiration of $\phi$ on velocity.

**Figure 9:**
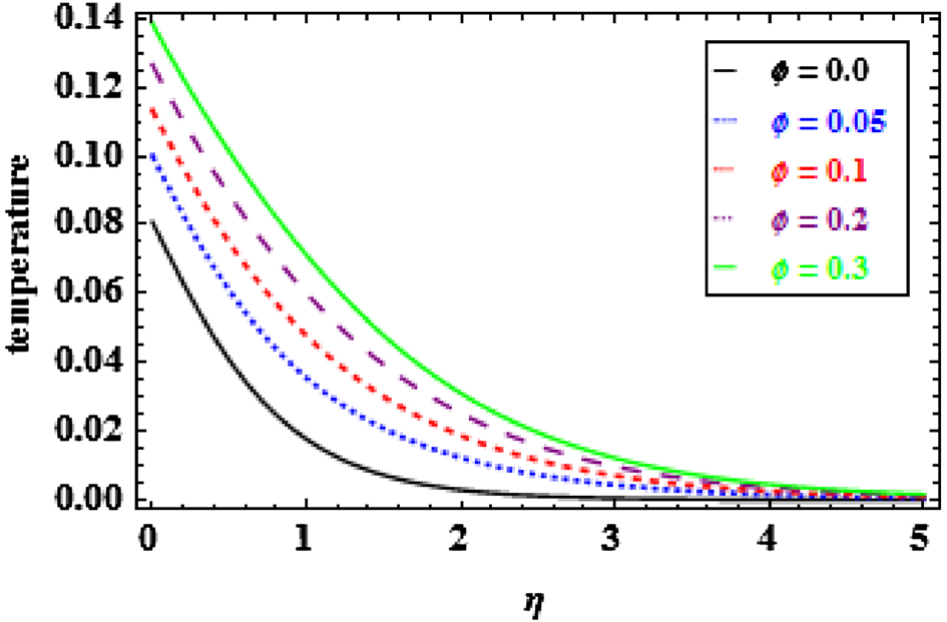
Inspiration of $\phi$ on temperature.

The influence of the slip parameter $A$ on flow and heat is seen in Figs. 3 and 4. With the increasing values of $A$ rise, the momentum boundary layer thickness (MBLT) declines, which results in a drop in fluid flow. Because as the slip factor upsurges, the slip at the surface wall increasing, this results in less surface penetration into the fluid. Figures 5 and 6 show the variance in heat and velocity outlines for various porous factor ${P}_{r}$ values. It is evident that the prevalence of porous media escalations the flow restriction, which decelerates the fluid movement. As a result, the resistance to fluid motion increases with an increase in the permeability factor, as a consequences reduction in velocity is observed. Figure 6 illustrates the consequence of growing permeability parameter ${P}_{r}$ contributes to the thermal boundary layer thickness (TBLT). It is analyzed that the absorbent medium resists the fluid movement. Due to this resistance the fluid motion decreases which increases the fluid temperature. Figure 7 depicts the impact of Biot number $Bn$ on the heat distribution for stable values of $Pr = 10, Nr = 1.0,$ and ${\theta }_{r}=1.5$. It demonstrates that when the values of $Bn$ are enhanced the heat inside the fluid enhances. Here, it is to be prominent that the TBLT enhances due to the convectively heat exchange at the sheet. In compared to the constant surface temperature circumstances, the NF with a convective boundary condition serves as a more relevant model. The consequence of volume fraction factor $\phi$ on the heat and velocity outlines is seen in Figs. 8 and 9. These graphs indicate that the velocity falls through the boundary layer area as the values of $\phi$ are enhanced. Physically, as the concentration of nanomaterials increases develops the friction in the flow, due to which the velocity of fluid drops.

**Figure 10:**
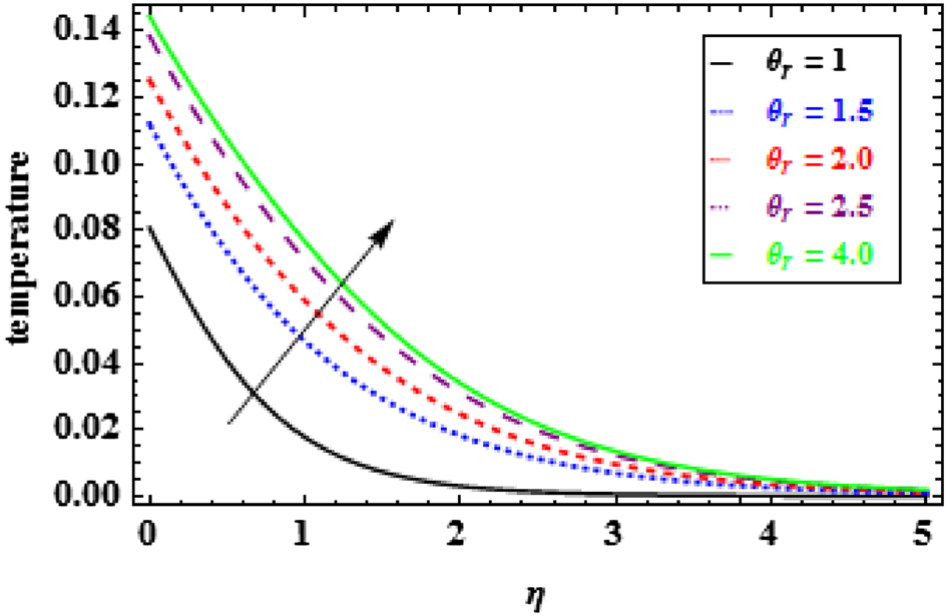
Inspiration of ${\theta }_{r}$ on temperature.

**Figure 11:**
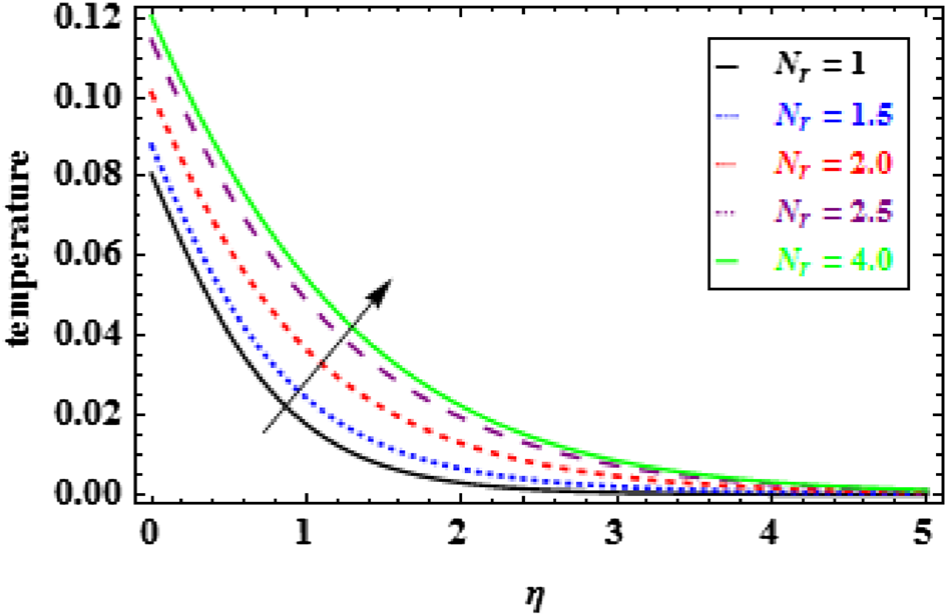
Inspiration of ${N}_{r}$ on temperature.

**Figure 12:**
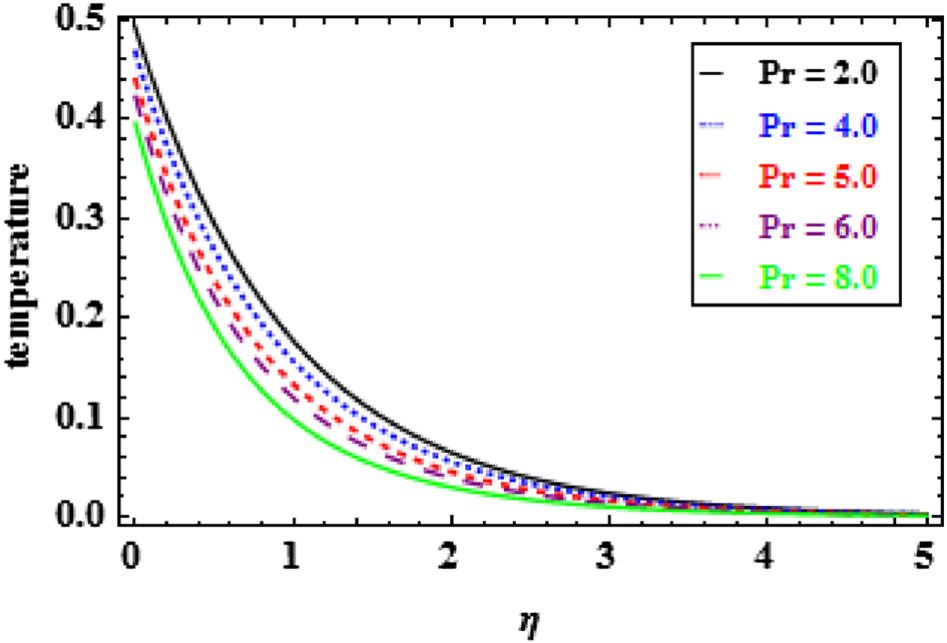
Inspiration of $Pr$ on temperature.

The variation of temperature distribution for various values of $\phi$, is explored in Fig. 9. The thermal efficiency increases with the increasing values of $\phi$, which causes an escalation in the TBLT. The influence of ${\theta }_{r}$ on heat profiles is noticed in Fig. 10. One can see from this figure that when the temperature ratio parameter rises, the fluid becomes more thermally stable, which raises the temperature profiles. Figure 11 shows how the radiation parameter $Nr$ affects the temperature. A significant result is found that as $Nr$ rises, the temperature profile enhances. As a consequence the fluid become heated, which enhance the temperature and TBLT. The variation of temperature profiles is depicted in Fig. 12 for dissimilar amounts of Prandtl number $Pr$. From this figure, it is witnessed that as *Pr* growths, the TBLT decrease significantly. As a result, the wall temperature gradient tends to increase. Physically, as the Prandtl number enhances the thermal conductivity declines and a result the TBLT decreases as a consequence the temperature distribution decreases. It is noted that samall $Pr$ denotes lower thermal conductivity fluid which develops large TBLT phenomena compared to higher $Pr$.

**Figure13:**
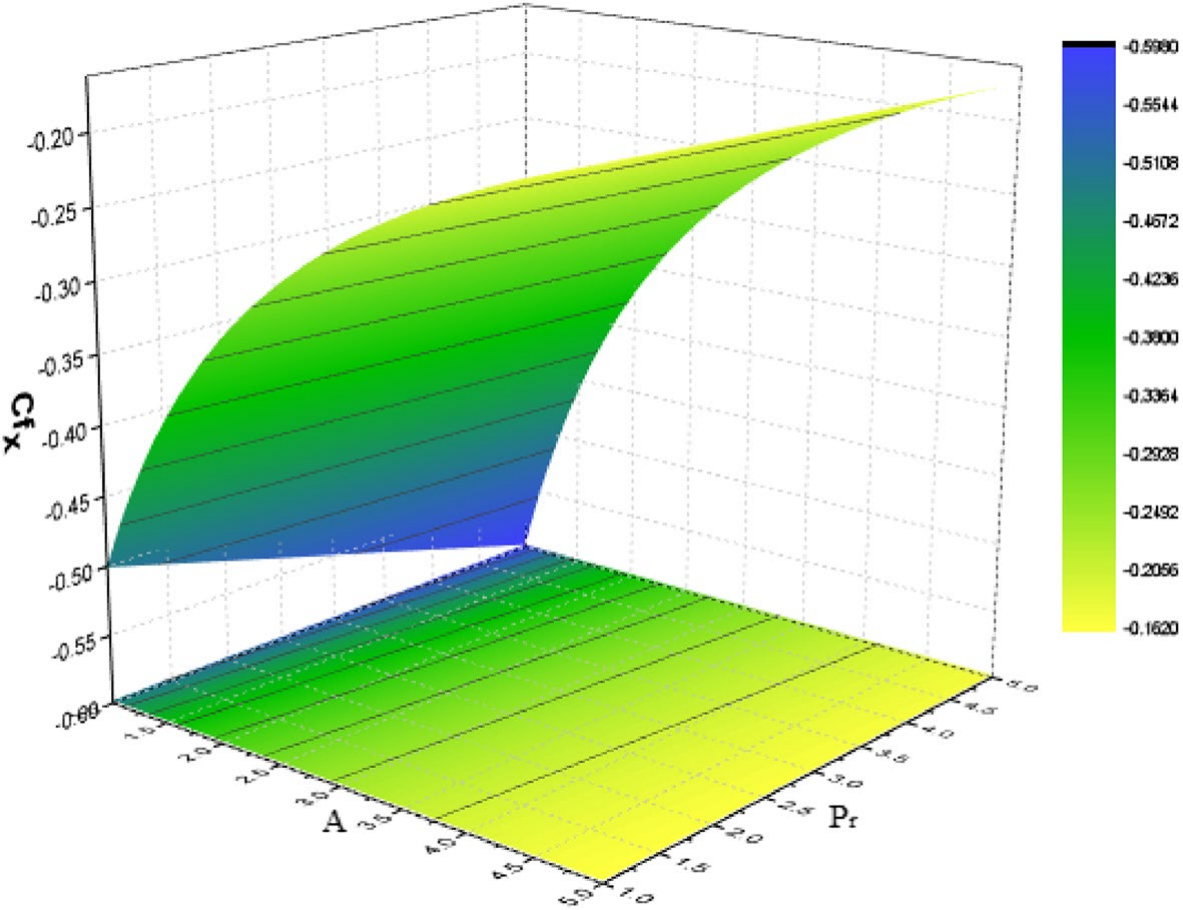
Effect of $A$ and ${p}_{r}$ on SF.

**Figure 14:**
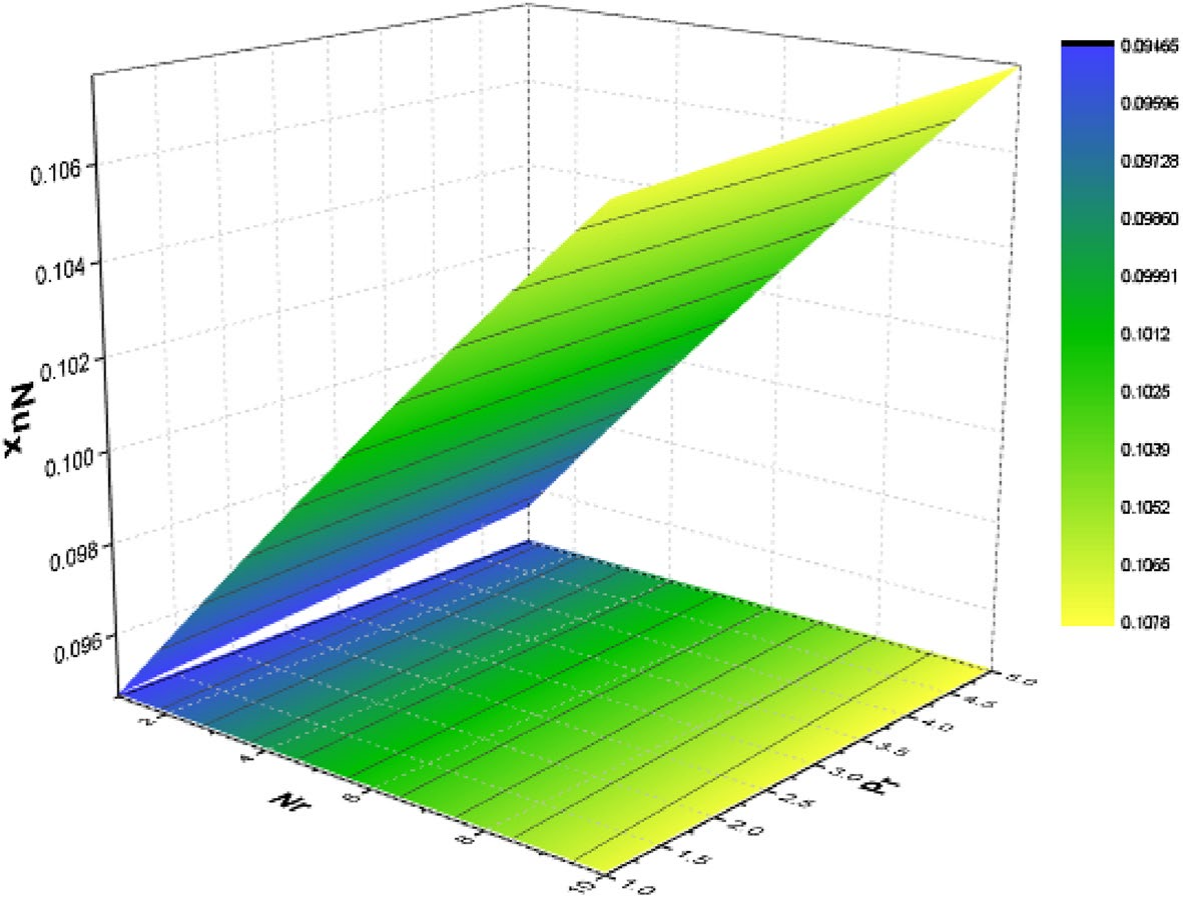
Effect of $Nr$ and $Pr$ on NN.

**Figure 15:**
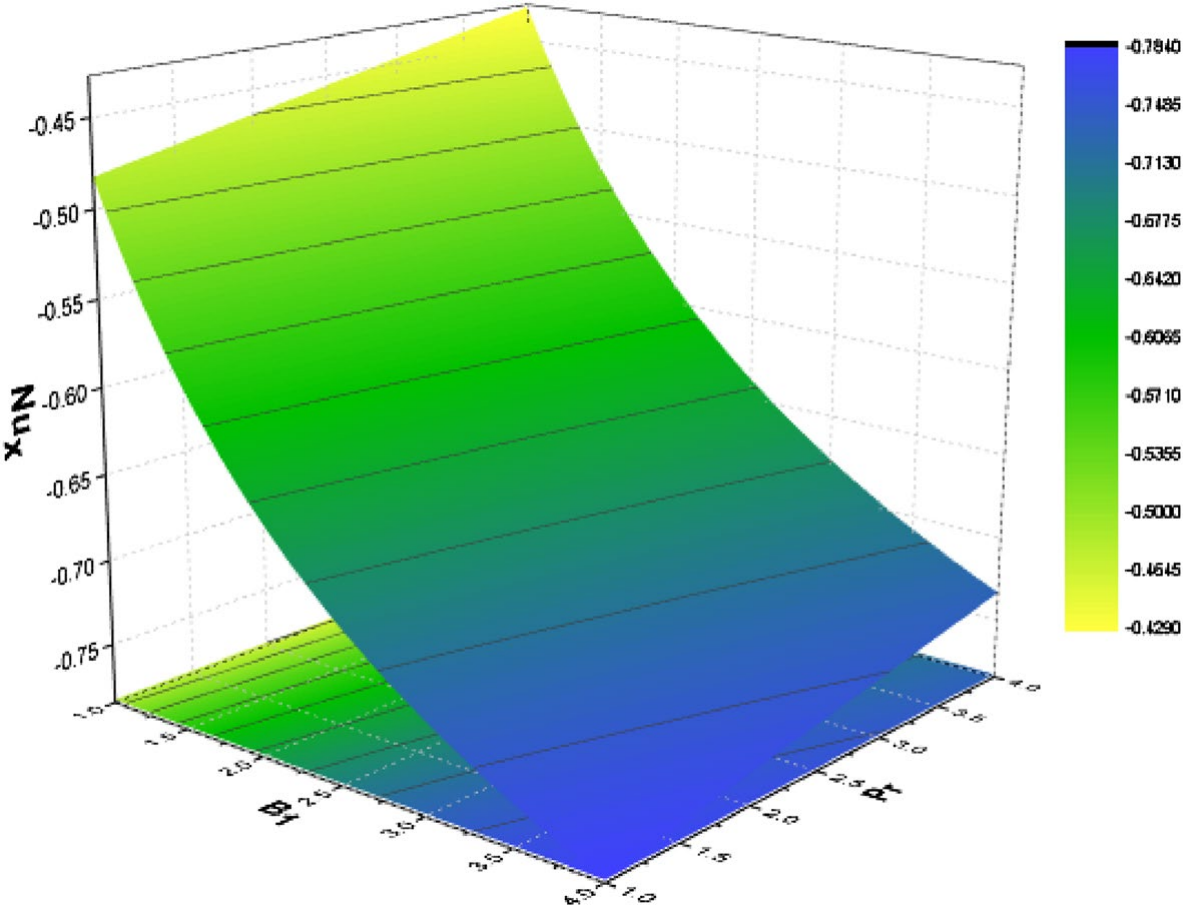
Influence of $Pr$ and $Bn$ on the NN.

For various types of nanoparticles, the variation of some physical factors ${C}_{f}$ (Local Skin Friction) and Nu (NN) are shown in Figs. 13, 14, 15 with the variation of the A, $kp, Nr, Pr$ and $Bn$. It is observed that as the magnitudes of $A$ and ${p}_{r}$ are increased the SF enhances as shown in Fig. 13. The parameters $Nr, Pr,$ and $Bn$ have significant effect on the NN which shows enhances in the NN as depicted in Figs. 14 And 15 respectively.

**Figure 16:**
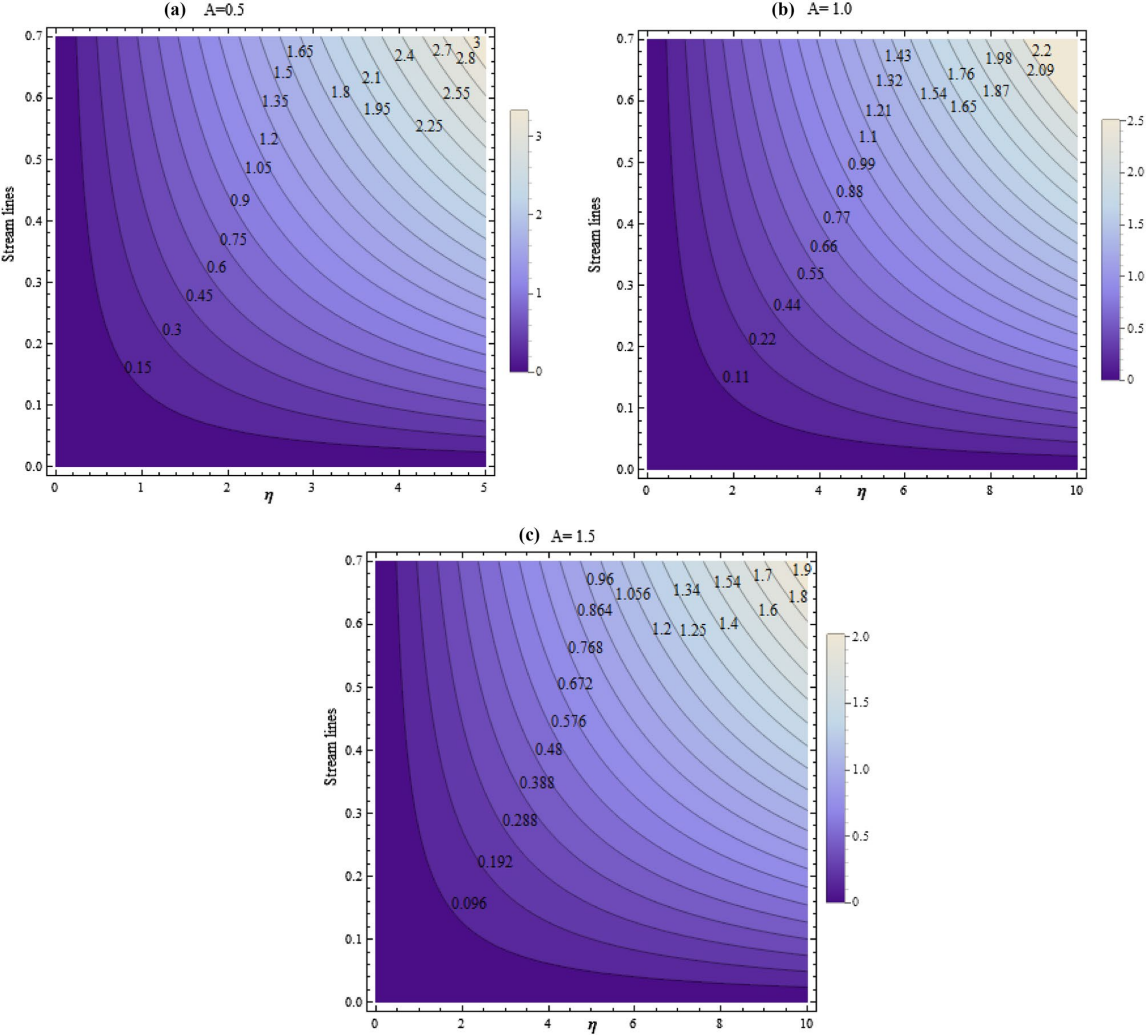
Streamlines for numerous values of A.

**Figure 17:**
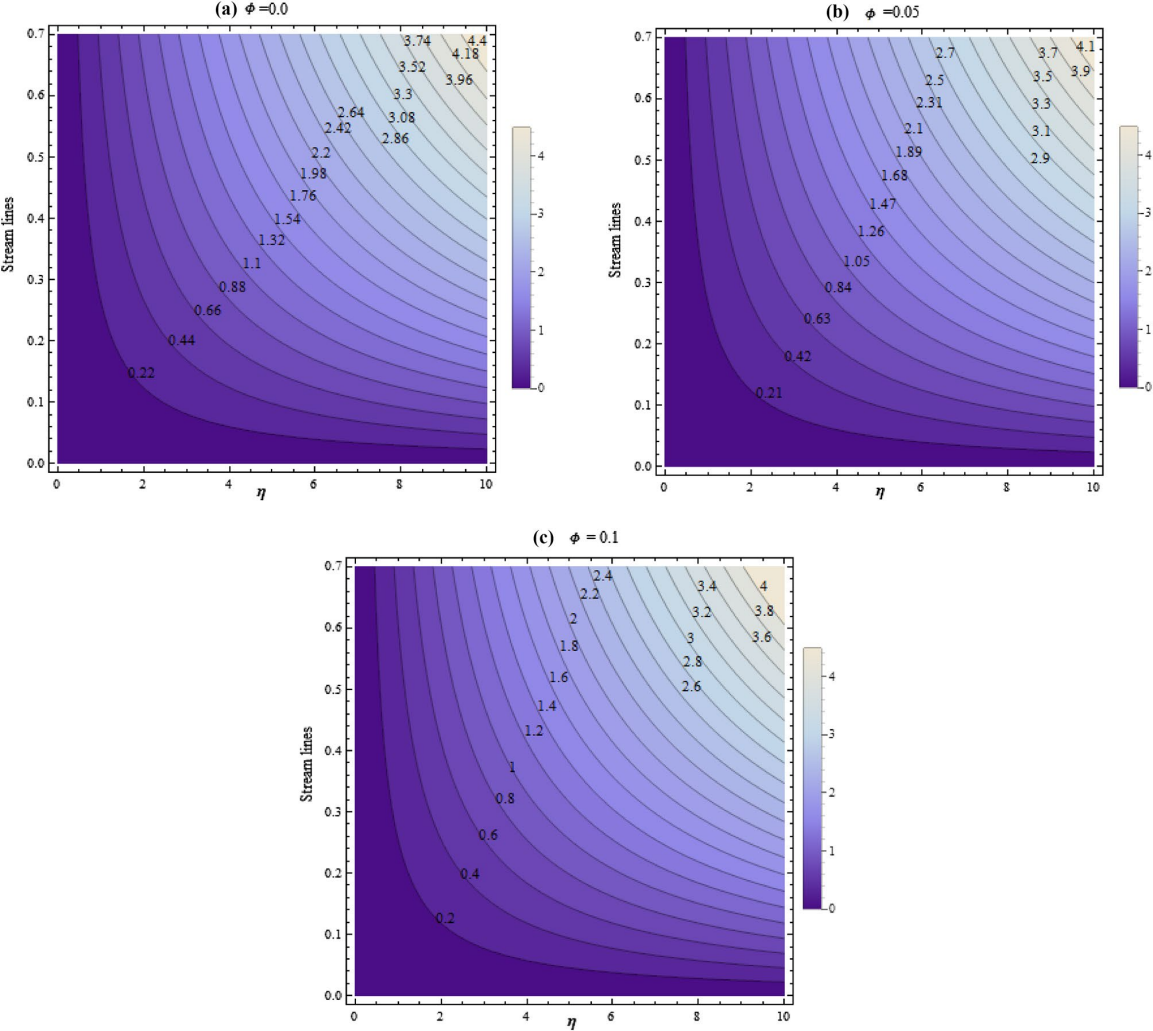
Streamlines for numerous values of $\phi$.

Morever, the Figs. 16a–c and 17a–c shown the streamlines for numerous values of $A$ and $\phi$. Figure 16a–c depicts that the stream lines drops with the growing values of $A$. It is found that stream lines declines with the growing values of $\phi$ as revealed in Fig. 17a–c.

For several kinds of water-based nanoparticles, calculations of the SFC (Skin Friction coefficient) are shown in Table 3. The SFC is discovered to increase with ${P}_{r}$ and $\phi$. Additionally, the slip parameter causes the friction factor to decrease. Additionally, the wall shear stress is lowest for NF containing Al_2_O_3_ and highest for those containing *Cu*. Table 4 lists the heat transfer rate caused by the heat variation at the wall for some physical factors including slip, temperature factor, volume fraction, radiation, porosity as well as Biot number. It has been shown that the heat transmission reduces with A and ${p}_{r}$ while increasing with $\phi$, ${\theta }_{r}$, *Nr*, *Pr*, and *Bn.* It is noted that TiO_2_ nanoparticles obtain the lowermost heat transfer rate, which is obvious given that TiO_2_ has the lowest thermal conductivity as comparison towards other nanoparticles.

**Table 3:** Variation of Skin friction for water-based nanomaterial.

A	${p}_{r}$	$\phi$	${\mathrm{Al}}_{2}{\mathrm{O}}_{3}$	$\mathrm{Cu}$	${\mathrm{TiO}}_{2}$
0			2.1882	2.5715	2.2117
0.2			1.3128	1.4179	1.3196
1.2			0.9759	0.9256	0.9792
	0		1.1155	1.2719	1.1263
	0.5		1.4548	1.5321	1.4597
	2		1.6554	1.6938	1.6583
		0	0.7993	0.7993	0.7993
		0.2	0.9193	0.9719	0.9226
		0.3	1.3128	1.4179	1.3196

**Table 4:** Disparity of the Nusselt number for numerous values of water-base nanomaterial.

A	${p}_{r}$	$\phi$	${\theta }_{r}$	${N}_{r}$	$Pr$	$Bi$	${\mathrm{Al}}_{2}{\mathrm{O}}_{3}$	$\mathrm{Cu}$	${\mathrm{TiO}}_{2}$
0							1.3731	1.3995	1.2436
0.2							1.2997	1.3126	1.1814
1.2							1.2479	1.2535	1.1367
	0						1.3532	1.3532	1.2157
	0.5						1.2739	1.2739	1.1493
	2						1.1932	1.1932	0.9897
		0					0.6977	0.6977	0.7972
		0.3					0.9519	0.9519	1.1814
		0.3					1.3126	1.3126	1.1814
			1.1				1.2997	1.3126	1.1814
			1.4				1.9529	1.9594	1.8617
			2.0				3.9761	3.9212	3.5975
				1			0.9493	0.9647	0.9539
				2			1.5338	1.5398	1.3943
				4			1.9555	1.8485	1.7819
					5		1.1699	1.1656	0.9719
					7		1.2478	1.2521	1.1369
					10		1.2997	1.3126	1.1814
						0.1	1.2997	1.3126	1.1814
						4	3.6179	3.4229	3.3927
						8	3.7393	3.5269	3.5128

## Conclusion

A numerical analysis using bvph2 built-in function in MATLAB for the investigation of water based NF containing Al_2_O_3_ nanoparticles over the permeable stretched sheet is investigated along with nonlinear TR of boundary layer flow with convective boundary conditions. Basic equation of flow are first converted to ordinary first order differential equation through by introducing new variables and then solved numerically. The influence of emerging parameters on the velocity, temperature, Skin Friction and Nussetl number are investigated using graphs and tables. The streamlines are also shown in this analysis. We observed the following conclusions:

- The TBLT increases as the temperature and radiation factors are increased.
- It is perceived that the velocity outline decays with the growing values of volume fraction factor.
- When the permeability and velocity slip parameters are improved the velocity distribution enhances.
- It is investigated that the temperature inside the fluid enhances as the values of velocity slip factor, permeability factor and Biot number is enhanced.
- For the growing values of ${\theta }_{r}$, $\phi$, and $Nr$, the temperature is enhances.
- It is detected that the velocity slip factor causes the friction factor to decrease.
- Additionally, the wall shear stress is lowest for NF containing Al_2_O_3_ and highest for NF containing Cu.

## Data Availability

The datasets generated and/or analyzed during the current study are not publicly available but are available from the corresponding author on reasonable request.

## Abbreviations

$\rho_{nf}$: Density of the fluid
$$q_{r}$$: Thermal radiation
$${P}_{r}$$: Permeability parameter
TBLT: Thermal boundary layer thickness
$\mu_{nf}$: Viscosity of NF
${q}_{w}$: Surface heat flow
*A*: Slip factor
${\rho }_{f}$: Density of the base fluid
$$\alpha_{nf}$$: Thermal conductivity
$\tau_{w}$: Surface shear stress
$$Bn$$: Biot number
$$\sigma *$$: Electrical conductivity
$\left( {\rho Cp} \right)_{nf}$: Heat capacity of NF
${C}_{f}$: Skin friction coefficient
$$f^{\prime}$$: Dimensionless velocity
${(\rho {C}_{p})}_{nf}$: Heat capacity of the nanoparticle
${\mu }_{f}$: Viscosity of fluid
$N{u}_{x}$: Local Nusselt number
$$\phi $$: Dimensionless concentration
${T}_{f}$: Temperature of the fluid
$$Pr$$: Prandtl number
$$\theta $$: Dimensionless temperature
$\theta_{r} = \frac{{T_{f} }}{{T_{\infty } }},\theta_{r} > 1$: Thermo ratio parameter
${\text{Re}}_{x} = \frac{{ax^{2} }}{{\nu_{f} }}$: Local Reynolds number
$$Nr$$: Radiation parameter
